# High-Dose Vitamin D Supplementation Improves Microcirculation and Reduces Inflammation in Diabetic Neuropathy Patients

**DOI:** 10.3390/nu12092518

**Published:** 2020-08-20

**Authors:** Tatiana Karonova, Anna Stepanova, Anna Bystrova, Edward B. Jude

**Affiliations:** 1 Almazov National Medical Research Centre, Institute of Endocrinology, 2 Akkuratova str., 197341 St. Petersburg, Russia; bystrova@inbox.ru; 2 Internal Medicine Department, Pavlov First Saint Petersburg State Medical University, 6-8 L.Tolstoy str., 197022 St. Petersburg, Russia; annstepanova12@gmail.com; 3 Tameside Hospital NHS Foundation Trust, Ashton Under Lyne OL69RW, UK; Edward.jude@tgh.nhs.uk

**Keywords:** diabetes, neuropathy, microcirculation, 25(OH)D, vitamin D, inflammatory markers

## Abstract

We assessed the effect of different doses of vitamin D supplementation on microcirculation, signs and symptoms of peripheral neuropathy and inflammatory markers in patients with type 2 diabetes (T2DM). Sixty-seven patients with T2DM and peripheral neuropathy (34 females) were randomized into two treatment groups: Cholecalciferol 5000 IU and 40,000 IU once/week orally for 24 weeks. Severity of neuropathy (NSS, NDS scores, visual analogue scale), cutaneous microcirculation (MC) parameters and inflammatory markers (ILs, CRP, TNFα) were assessed before and after treatment. Vitamin D deficiency/insufficiency was detected in 78% of the 62 completed subjects. Following treatment with cholecalciferol 40,000 IU/week, a significant decrease in neuropathy severity (NSS, *p* = 0.001; NDS, *p* = 0.001; VAS, *p* = 0.001) and improvement of cutaneous MC were observed (*p* < 0.05). Also, we found a decrease in IL-6 level (2.5 pg/mL vs. 0.6 pg/mL, *p* < 0.001) and an increase in IL-10 level (2.5 pg/mL vs. 4.5 pg/mL, *p* < 0.001) after 24 weeks of vitamin D supplementation in this group. No changes were detected in the cholecalciferol 5000 IU/week group. High-dose cholecalciferol supplementation of 40,000 IU/week for 24 weeks was associated with improvement in clinical manifestation, cutaneous microcirculation and inflammatory markers in patients with T2DM and peripheral neuropathy.

## 1. Introduction

It is well known that vitamin D deficiency along with type 2 diabetes mellitus (T2DM) is a modern pandemic [1,2]. The development of microvascular complications in T2DM worsens both the prognosis and the patients’ quality of life. There is increasing evidence of a possible contribution of vitamin D deficiency to the pathogenesis of diabetes and its complications [3]. Large-scale studies have shown 40% increased risk of developing diabetes in individuals with a reduced 25(OH)D (25-hydroxy vitamin D) level [4], as well as 24% decrease in diabetes risk for every 25 nmol/L increase in 25(OH)D concentration [5]. However, some studies found no association between diabetes risk and vitamin D status [6]. Thus, a recent interventional prospective study demonstrated no decrease in the risk of T2DM development in patients with prediabetes after two-year treatment with 4000 IU of vitamin D per day [7]. However, some experts suggested that 4000 IU is not a sufficient supplementation dose for patients with already existing impaired glucose metabolism, and besides, most study participants had normal basal 25(OH)D levels [8]. These results do not exclude the presence of pleiotropic vitamin D effects on insulin secretion, insulin resistance and adipocytokine system [9,10,11,12], and the possibility of influencing the development of microvascular diabetic complications [3]. Along with immune-mediated mechanisms, microcirculation deterioration in patients with diabetes has been found to play an important role in the pathogenesis of microvascular complications, including peripheral neuropathy (DPN) [12,13].

Vitamin D deficiency is also believed to play a role in the progression of DPN [14,15,16]. One study showed that vitamin D supplementation in patients with T2DM and DPN resulted in the pain decrease and reduction or withdrawal of semisynthetic opioids, and that an increase in 25(OH)D by 1 ng/mL was associated with the decrease in neuropathy severity and increase in impulse conduction frequency along nerve fibres by 2.2% and 3.4%, respectively [15]. Another study demonstrated a relationship between serum 25(OH)D levels and the severity of neuropathy in T2DM, where the greatest changes were found in patients with 25(OH)D levels of less than 16 ng/mL [16]. Possible association of vitamin D deficiency with DPN was revealed by other investigators, but relationships between 25(OH)D level and DPN remain unclear [17]. The Cochrane systematic review demonstrated no convincing evidence regarding vitamin D effectiveness in chronic painful conditions [18]. Some studies found high levels of IL-13 and IL-17 in patients with T2DM and DPN, and negative correlations between these interleukins and 25(OH)D levels [19].

Thus, the correction of vitamin D deficiency in patients with T2DM is becoming increasingly attractive for the prevention and treatment of microvascular complications. However, the question of the required vitamin D dose and the treatment duration remains highly debatable. According to some studies, the daily dose of vitamin D for pleiotropic effects should exceed the dose recommended for prophylaxis [20,21]. The inconsistency of the evidence dictates the need for further research in this field.

The aim of this study was to assess the effect of therapy with different doses of cholecalciferol for 24 weeks on parameters of microcirculation, clinical manifestations of peripheral neuropathy and inflammatory markers in patients with T2DM.

## 2. Materials and Methods

### 2.1. Study Population

We conducted a prospective randomized trial in patients with T2DM and DPN. Ninety-eight patients with T2DM and DPN were screened for the study from January 2018 to January 2019. Patients were selected based on the following inclusion criteria: (i) Males and females with T2DM aged 18 to 65 years; (ii) diabetes duration ≥5 years; (iii) HbA1c <9%; (iv) stable hypoglycaemic, hypotensive and hypolipidemic therapy; and (v) neurological deficit of 4 points or more according to the neuropathy disability score (NDS). Exclusion criteria were as follows: Current and former smokers, obliterating atherosclerosis, diabetic foot or Charcot osteoarthropathy, inflammatory joint diseases, B12 deficiency, vitamin D supplementation, treatment with tricyclic antidepressants, anticonvulsants, opiates or nonsteroidal anti-inflammatory drugs. The patient’s decision against further participation in the trial, failure to appear at the scheduled time and any acute inflammatory/infectious disease during the trial were withdrawal criteria.

**Figure 1:**
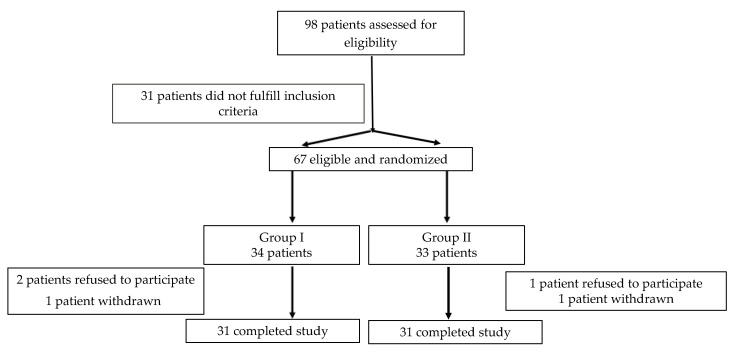
Flowchart showing patient randomization and disposition.

Sixty-seven patients (34 females, median age 56 (49; 61) years) were enrolled into the study. Patients were randomized using the even/odd method into two cholecalciferol treatment groups: Group I (*n* = 34) 5000 IU once weekly and Group II (*n* = 33) 40,000 IU once weekly, taken orally for 24 weeks. Three patients refused to participate in the study after randomization. Two patients developed upper respiratory tract infection and were withdrawn from the study soon after randomization. Thus, 62 patients completed the study (31 patients from each group; Figure 1).

The trial was performed at the First Pavlov State Medical University and Almazov National Medical Research Centre, St. Petersburg, Russia, and it was conducted in compliance with the principles of the Declaration of Helsinki. Each patient gave written informed consent before enrolment. The study was approved by the local ethics committee.

### 2.2. Data Collection

Patient demographics (gender, age, height, body weight, calculated body mass index (BMI)) and blood pressure (BP), anamnesis (diabetes duration, complications, concomitant diseases, medications, smoking and alcohol intake) and anthropometric data were assessed at baseline.

Neuropathy assessment was done using standard tests: NSS (neuropathic symptomatic score) [22], NDS (neuropathic disability score) [23] and VAS (visual analogue scale, to measure painful symptoms) [24].

Laboratory tests were performed before and 24 weeks after cholecalciferol treatment. Blood samples were taken from the antecubital vein in the morning after an overnight fast (not less than 12 h after the last meal) and centrifuged at 4000 rpm and serum was stored at −20 °C until analysis.

Serum total cholesterol (TC, reference values 3.5–5.0 mmol/L) and C-reactive protein (CRP, reference values 0.00–5.00 mg/L) levels were evaluated on automatic biochemical analyser (COBAS INTEGRA 400 plus, Roche Diagnostics GmbH, Mannheim, Germany). Determination of HbA1c (reference values 4.0–6.0%) was carried out on a Bio-Rad D-10 Chemistry Analyzer (Bio-Rad Diagnostics, Hercules, USA). Serum 25(OH)D level was measured by chemiluminescent immunoassay with commercial laboratory kits and control kits (Abbott Laboratories, Waukegan, USA) using an Architect i2000 analyser (Abbott, Abbott Park, IL, USA). Vitamin D deficiency was defined as serum 25(OH)D level < 20 ng/mL, insufficiency—from 20 ng/mL to 30 ng/mL and adequate vitamin D level > 30 ng/mL [25]. The level of parathyroid hormone (PTH, reference values 15.0–65.0 pg/mL) was estimated using chemiluminescent immunoassay on microparticles (Architect i2000, Abbott, Abbott Park, IL, USA). Serum interleukins (IL) and tumour necrosis factor-α (TNFα) were determined by enzyme-linked immunosorbent assay (Bio-Rad 680 Microplate Reader, Hercules, USA) using the appropriate sets of reagents for enzyme immunoassay to determine the concentration of IL-1β (reference values 0–5.0 pg/mL), IL-6 (reference values 0–7.0 pg/mL), IL-10 (reference values 0–9.1 pg/mL) and TNFα (reference values 0–8.21 pg/mL) (Vector-Best, Novosibirsk, Russia).

Skin microcirculation (MC) was assessed at baseline and after 24 weeks of vitamin D therapy by the laser Doppler flowmetry (LDF) method (LAKK-M complex, LAZMA LLC, Moscow, Russia) using standard functional tests (occlusal and orthostatic). LDF measurements were carried out at room temperature of 24 °C. Basal MC was evaluated on the plantar surface of the big toe of the right lower limb in supine position after 15-min rest, during which the test area was not covered [26]. The average MC parameters measured in perfusion (pf) units were automatically calculated: M—average perfusion value, σ—average blood flow modulation, Kv—coefficient of variation (%). Post occlusal (Mbase—average value of MC before occlusion (pf unit); Moccl—indicator of MC in the process of occlusion (“biological zero”) (pf unit); Mmax—maximum value of MC during the postocclusal hyperemia (pf unit); RCB—reserve of capillary blood flow (the ratio of Mmax to Mbase, %) and orthostatic LDF tests (Mbase average value of MC before orthostasis (pf unit), Mmin (pf unit) —minimal decrease in blood flow, and the degree of decrease in blood flow (DDB) (%)) were performed for each diabetic patient. In healthy subjects, RCB ranged from +80 to +150%, and the normal decrease in the level of MC during the postural test reached 30–45%.

### 2.3. Study Objective

The primary outcome was to evaluate the effect of high and low dose of vitamin D on skin microcirculation after 24 weeks of treatment. The secondary outcomes were change from baseline in plasma interleukins and TNFα at 24 weeks. Other secondary outcomes were change in clinical and symptom scores for neurological status (NDS, VAS and NSS), all assessed at baseline and at 24 weeks.

### 2.4. Statistical Analysis

Statistical data processing was carried out using the licensed software package SAS 9.4 (SAS, Buckinghamshire, UK). All data points for both treatment groups were collected, hence data imputation was not implemented in this study. Results are presented as median and interquartile range [IQR, Q25; Q75]. Comparison of the indicators in the groups before and after treatment was performed by Wilcoxon T-test. Parameters of the two treatment groups were compared using Mann–Whitney U-test. Analysis of clinical and laboratory data was evaluated using the Pearson χ^2^ criterion. The relationship between the indicators was assessed by the Spearman rank correlation method. Statistical significance was defined as *p* < 0.05.

Clinical Trial Registration: URL: https://clinicaltrials.gov. Unique Identifier: NCT04377399.

## 3. Results

The basic characteristics of the study participants are presented in Table 1. The median age of the study participants was 56 (49; 61) (range 36 to 65) years, BMI—30.2 (28.3; 32.7) kg/m^2^, serum 25(OH)D—17.2 (10.2; 27.9) ng/mL, HbA1c—7.9 (7.2; 8.4)%. Both treatment groups were matched for age, gender, diabetes duration, BMI, HbA1c, neuropathy severity, comorbidities and concomitant medications. Glucose-lowering and concomitant therapy was stable throughout the study period.

At the beginning of the study most patients had vitamin D deficiency/insufficiency (25 patients (79.7%) from group I; 24 patients (77.4%) from group II). After 24 weeks of cholecalciferol intake, an increase in serum 25(OH)D was observed in both groups. Thus, all patients taking 40,000 IU per week reached 25(OH)D levels of ≥30 ng/mL after 24 weeks of treatment, while only 15 patients (48.4%) from group I (5000 IU weekly) achieved a normal vitamin D value.

After 24 weeks of treatment, a significant decrease in BMI (*p* = 0.001), HbA1c level (*p* = 0.004), serum IL-6 (*p* < 0.001) and an increase in serum IL-10 (*p* < 0.001) were found in patients taking 40,000 IU of cholecalciferol per week. Weight loss of more than 5% was seen in 19 patients (61%) from this group. No significant changes of any of the above parameters were observed in group I. Both groups showed no significant changes in total cholesterol, PTH, IL-1β, TNFα and CRP after 24 weeks of treatment. Baseline and follow-up values of investigated parameters are presented in Table 2.

At the end of the study, a negative correlation between the level of 25(OH)D and HbA1c (*r* = −0.388, *p* = 0.031) and positive correlation of HbA1c with IL-6 (r = 0.426, *p* = 0.017) and IL-10 (*r* = −0.391, *p* = 0.030) was observed in Group II. Also, the correlation analysis revealed an interlink between the severity of neurological deficit and HbA1c level (*r* = −0.352, *p* = 0.003).

Baseline parameters of MC (M, σ, and Kv) did not differ between the treatment groups (p_MI-II_ = 0.08; p_σI-II_ = 0.08; p_KvI-II_ = 0.74). After 24 weeks of treatment, a significant difference between the initial and final Kv (*p* < 0.001) was found only in Group II. This increase in Kv reflects an improvement in microcirculation in patients taking 40,000 IU cholecalciferol per week. Correlation analysis revealed a significant relationship between final levels of 25(OH)D and Kv (*r* = 0.51; *p* = 0.04) in patients from Group II. No associations and significant changes were detected in Group I (Table 2). The postural test demonstrated a significant increase in DDB after 24 weeks of treatment (*p* < 0.001) that was detected only in Group II. After 24 weeks of treatment, M_max_ increased significantly in both groups of patients (*p* = 0.012; *p* = 0.003). There were no differences between the initial and final RCB in Group I (*p* = 0.056), but a significant increase in RCB was found in Group II (*p* < 0.001). Indicators of skin microcirculation before and after 24 weeks of vitamin D therapy are presented in Table 3.

Initially, all patients had neurological deficit of more than 4 points according to the neuropathy disability score (NDS). The median severity of neurological deficit was 8, which corresponds to moderately severe diabetic neuropathy. No differences in neuropathy manifestation evaluated by NSS and VAS were observed between the groups. After 24 weeks of treatment, patients from Group II (40,000 IU/week) demonstrated a significant decrease in neurological deficit (NDS points decreased from 8 to 6, *p* = 0.001), reduction of pain severity assessed by VAS (from 50 (42.5; 55) mm to 47 (37.5; 51) mm, *p* = 0.001), and significant decrease in neuropathic symptomatic score points (from 5 (4; 6) to 4 (4; 5), *p* = 0.001). No changes were found in Group I (5000 IU/week).

## 4. Discussion

Vitamin D deficiency is widespread throughout the world, and patients with obesity, prediabetes, gestational diabetes and T2DM constitute a high-risk group [9,27,28,29]. Given the presence of obesity in most subjects with impaired glucose metabolism, prophylactic doses of vitamin D for this population should be significantly higher than for individuals with normal body weight [25,27,30]. Our study revealed a very high prevalence of vitamin D deficiency/insufficiency in patients with T2DM, which is consistent with previously reported data [6,17]. After six months, all patients taking 40,000 IU of cholecalciferol per week achieved normal vitamin D levels, while only half of the patients receiving 5000 IU weekly reached normal 25(OH)D concentration. This finding suggests the need to prescribe higher doses of vitamin D for patients with T2DM.

Interestingly enough, vitamin D supplementation has been reported to be associated with body weight reduction, decrease in HbA1c and insulin resistance and improvement in insulin sensitivity [28,29]. Also, patients with higher baseline vitamin D levels have a greater degree of weight loss than those with lower baseline 25(OH)D level [31]. After 24 weeks of treatment, our study found a negative correlation between serum 25(OH)D and BMI in patients receiving 40,000 IU of cholecalciferol weekly, which was 5714 IU/day. We also found a decrease in HbA1c level in patients in group II (40,000 IU/week), though no change in diabetes treatment was introduced. Whether it was an independent vitamin D effect or mediated through body weight reduction remains to be determined. Our results support previously demonstrated correlation between increase in serum 25(OH)D and decrease in HbA1c in patients with T2DM [5,20].

Another fact we know is that chronic microvascular complications in T2DM lead to early disability and significantly increase the cost of treatment [32]. Vitamin D deficiency has been shown to affect diabetic complications by influencing glucose metabolism and inflammatory process [3,33]. Pleiotropic effect of vitamin D on inflammation has been found to play an important role in DPN development, and it is of great scientific and practical interest [34]. Some studies have shown higher TNFα and lower IL-10 levels associated with increased HbA1c in patients with T2DM and DPN than in patients with impaired glucose tolerance and healthy controls [35].

Regarding CRP, its concentration is considered to be a surrogate marker of inflammation, and its increase in T2DM has been also discussed [36]. Thus, in patients with metabolic syndrome, vitamin D therapy resulted in significant IL-6 reduction but did not change CRP concentration [37]. The REGARDS study showed an association between low serum 25(OH)D and increase in IL-6 and CRP levels, and found no associations with IL-10 [38]. Meta-analysis of 20 randomized clinical trials demonstrated lower levels of CRP and TNFα and no differences in IL-6 concentration in patients taking vitamin D therapy compared to the control group [39]. At the same time, active forms of vitamin D have been shown to reduce TNFα and IL-6 production and stimulate IL-10 production by immune cells [40]. The results of our study appeared to be consistent with the previously reported data concerning association between 25(OH)D levels and markers of inflammation, but a significant decrease in IL-6 and increase in IL-10 were revealed only in patients who received high-dose vitamin D therapy (40,000 IU per week) and reached normal 25(OH)D levels. Our findings suggest that normalization of serum 25(OH)D with high-dose cholecalciferol treatment affects inflammatory markers. It is known that immune cells have vitamin D receptors and can participate in the final stage of hydroxylation and in calcitriol formation [40]. An increase in 25(OH)D concentration with cholecalciferol treatment may contribute to activation of calcitriol synthesis, which, in turn, may influence release of proinflammatory and anti-inflammatory cytokines [39].

As for neuropathy, we found a decrease in neurological deficit and pain severity after 24 weeks of treatment with 40,000 IU of cholecalciferol weekly. We found no correlation between values of pain scales with serum 25(OH)D and ILs but found a correlation with HbA1c level. It can be assumed that the effect of cholecalciferol on peripheral nervous system in patients with T2DM and DPN was most likely mediated by improvement in metabolic parameters rather than resulting from direct vitamin D action.

Our study demonstrated significant improvement in skin microcirculation parameters only in patients receiving 40,000 IU of cholecalciferol per week. This effect can be explained by the direct protective action of vitamin D on endothelial cells through specific receptors [41], or it can be mediated by improvement in metabolic parameters and inflammatory status associated with high-dose therapy [42].

### Study Limitations

There is lack of data on 1,25-dihydroxyvitamin D (calcitriol) levels, which implements the main pleiotropic effects of vitamin D. There are several methods available to measure 25(OH)D levels. In this study, we used Abbott chemiluminescent immunoassay, which is the method available in our centre. Our study was an open-label one, so the possible effect of information about therapy on outcomes should be considered. We found a relationship between 25(OH)D and only some inflammatory markers, which makes further research in this area necessary. Most patients included in the study had vitamin D deficiency or insufficiency, so the effect of cholecalciferol therapy on peripheral neuropathy in patients with T2DM and normal 25(OH)D remains to be investigated. In addition, we want to highlight that since there is no gold standard for the assessment of microcirculation in diabetic patients, we chose Doppler flowmetry with two functional tests to perform this.

## 5. Conclusions

The study demonstrated that high-dose cholecalciferol therapy (40,000 IU/week) for 24 weeks resulted in 25(OH)D normalization and was associated with reduction in neuropathy severity, as well as improvement in skin microcirculation and cytokines profile (decrease in proinflammatory IL-6 and increase in anti-inflammatory IL-10), in patients with T2DM and DPN. Our findings suggest that vitamin D deficiency may be a modifiable factor which affects diabetic peripheral neuropathy and requires timely identification and correction with cholecalciferol at doses of more than 5000 IU/day. Further studies are needed to clarify the treatment duration and determine the optimal dose of vitamin D for patients with T2DM and DPN.

## Figures and Tables

**Table 1:** Baseline clinical characteristics of type 2 diabetic patients according to randomization.

Characteristics	5000 IU/Week, *n* = 31 (Group I)	40,000 IU/Week, *n* = 31 (Group II)	*p*
Males, *n* (%)/Females, *n* (%)	15 (48.4)/16 (51.6)	16 (51.6)/15 (48.4)	0.800
Age, years	57 (48; 62)	55 (52; 60)	0.756
Body mass index, kg/m^2^	30 (28.3; 31.8)	31 (29.5; 32.)7	0.155
Obesity, *n* (%)	21 (68)	20 (65)	0.789
Duration of type 2 diabetes, years	6 (5; 8.5)	7 (5; 11)	0.733
Diabetic peripheral neuropathy, *n* (%)	31 (100)	31 (100)	1.000
Neuropathic symptomatic score, points	5 (4; 6)	5 (4; 6)	0.799
Neuropathic dysfunctional score, points	8 (7; 9)	8 (7; 9)	0.857
Visual analog scale, mm	50 (40; 60)	50 (42.5; 55)	0.744
Diabetic retinopathy, *n* (%)	21(68)	24(77)	0.394
Diabetic nephropathy, *n* (%)	11(35)	9(29)	0.584
Arterial hypertension, *n* (%)	23 (74)	25 (81)	0.544
Coronary heart disease, *n* (%)	17 (55)	15 (48)	0.701
Insulin, *n* (%)	11 (35)	9 (29)	0.587
Metformin, *n* (%)	29 (94)	25 (81)	0.130
Sulfonylureas, *n* (%)	4 (13)	5 (16)	0.719
DPP-4 inhibitors, *n* (%)	5(16)	5(16)	1.000
SGLT-2 inhibitors, *n* (%)	1 (3)	3(10)	0.302
GLP-1R agonists, *n* (%)	-	1(3)	0.314
ACE inhibitors/ARB, *n* (%)	23 (74)	25 (81)	0.544
Calcium channel blockers, *n* (%)	5 (16)	7 (22)	0.521
β-adrenergic receptor blockers, *n* (%)	21 (68)	23 (74)	0.576
Diuretics, *n* (%)	14 (45)	11 (35)	0.438
Statins, *n* (%)	15 (48)	16 (52)	0.800

Data are presented as median, interquartile range [Q25; Q75] and percentages (%); DPP-4—Dipeptidyl-peptidase-4; SGLT-2—sodium-glucose transport protein 2; GLP-1R—Glucagon-Like Peptide-1 Receptor; ACE—angiotensin converting enzyme; ARB—angiotensin II receptor blockers.

**Table 2:** Anthropometric and laboratory parameters at baseline and after 24-week treatment.

Parameters	5000 IU/Week, *n* = 31 (Group I)		40,000 IU/Week, *n* = 31 (Group II)	
	Baseline	After 24 Weeks	Baseline	After 24 Weeks
BMI, kg/m^2^	30 (28.3; 31.8)	30 (28.4; 31.8)	31 (29.5; 32.7)	28,7 (25.4; 30.4) **^,#^
25(OH)D, ng/mL	18.8 (10.7; 27.4)	26.9 (20; 34.6) *	16.2 (8.7; 25.3)	71.6 (54.8; 88.3) **^,##^
HbA1c, %	7.9 (7.1; 8.3)	7.9 (7.2; 8.4)	7.9 (7.1; 8.5)	7.4 (6.5; 7.7) *^,#^
PTH, pg/mL	34.5 (24.3; 45.7)	28.6 (23.4; 40.4)	32.8 (23.5; 45.2)	26.6 (19.2; 34.6)
TC, mmol/L	4.9 (4.1; 6.1)	5.3 (4.1; 6.3)	5.5 (4.5; 6.5)	5.4 (4.7; 6.1)
TNFα pg/mL	2.0 (2.0; 2.0)	2.0 (2.0; 2.0)	2.0 (2.0; 2.0)	2.0 (2.0; 2.0)
CRP ml/L	1.4 (0.7; 2.0)	1.4 (0.8; 2.1)	1.5 (1.1; 2.0)	2.0 (0.8; 3.0)
IL-1β pg/mL	1.0 (1.0; 1.0)	1.0 (1.0; 1.0)	1.0 (1.0; 1.0)	1.0 (1.0; 1.0)
IL-6 pg/mL	1.9 (1.3; 3.1)	2.3 (1.3; 3.1)	2.5 (1.5; 4.1)	0.6 (0.5; 0.8) **^,##^
IL-10 pg/mL	3.3 (2.5; 4.8)	3.5 (2.5; 5.0)	2.5 (2.5; 3.6)	4.5 (3.5; 5.7) **^,#^

Data are presented as median and interquartile range (Q25; Q75); *p* value: * *p* < 0.05, ** *p* < 0.001—compared with previous results in the same group; ^#^ *p* < 0.05, ^##^ *p* < 0.001—between groups at baseline and after 24 weeks of therapy; BMI—body mass index; 25(OH)D—25-hydroxyvitamin D; HbA1c—glycated hemoglobin; PTH—parathyroid hormone; TC—total cholesterol; TNFα—tumor necrosis factor α; CRP—C-reactive protein; IL-1β—interleukin 1β; IL-6—interleukin-6; IL-10—interleukin-10.

**Table 3:** Microcirculation parameters at baseline and after 24-week treatment.

Parameters	5000 IU/Week, *n* = 31 (Group I)		40,000 IU/Week, *n* = 31 (Group II)	
	Baseline	After 24 Weeks	Baseline	After 24 Weeks
M, pf units	7.41 ± 3.97	7.16 ± 4.26 ^#^	6.01 ± 1.89	7.01 ± 2.46 *^,#^
σ, pf units	1.11 ± 0.57	1.05 ± 0.56 ^#^	0.85 ± 0.57	1.81 ± 1.14 *^,#^
Kν *, %	17.68 ± 10.14	18.89 ± 10.83 ^#^	16.65 ± 10.99	27.96 ± 16.38 *^,#^
Δ Kν, %	+6.8%		+68.3%	
***Postural Test***				
M_base_, pf unit	7.75 ± 1.8	7.78 ± 2.3 ^#^	6.69 ± 1.51	7.97 ± 2.13 *^,#^
M_min_, pf unit	6.10 ± 1.52	6.13 ± 2.26 ^#^	5.36 ± 1.47	5.07 ± 1.72 *^,#^
DDB, %	24.82 ± 9.27	23.87 ± 9.1 ^#^	23.4 ± 12.68	51.88 ± 36.71 **^,#^
Δ DDB, %	−3.8%		+121.7%	
***Occlusal Test***				
M_base_ pf unit	7.10 ± 1.72	6.74 ± 1.75 ^#^	6.49 ± 2.10	7.54 ± 2.89 *^,#^
M_max_ pf unit	9.73 ± 2.25	8.97 ± 3.60 ^#^	9.59 ± 3.15	14.57 ± 3.63 *^,#^
RCB, %	40.85 ± 20.31	35.79 ± 17.10 ^#^	48.57 ± 18.56	106.8 ± 44.8 **^,#^
Δ RCB, %	−12.4%		+120%	

Data are presented as median and interquartile range (Q25; Q75); *p* value: * *p* < 0.05—compared with previous results in the same group; ** *p* < 0.01—compared with previous results in the same group ^#^ *p* < 0.05—between groups after 24 weeks of therapy; M—average perfusion value; σ—average blood flow modulation; Kv—coefficient of variation (%); M_base_—average value of microcirculation before orthostasis or occlusion, M_min_—minimal decrease in blood flow; pf units—perfusion units; DDB—the degree of decrease in blood flow (%); M_max_—maximum value of microcirculation during the postocclusal hyperaemia; RCB—reserve of capillary blood flow (the ratio of M_max_ to M_base_, %); ∆—delta between baseline and 24 weeks parameters in the same group.
